# PD-L1 expression in PitNETs: Correlations with the 2022 WHO classification

**DOI:** 10.1016/j.bas.2024.104171

**Published:** 2024-12-24

**Authors:** Ethan Harel, Ekkehard Hewer, Stefano La Rosa, Jean Philippe Brouland, Nelly Pitteloud, Federico Santoni, Maxime Brunner, Roy Thomas Daniel, Mahmoud Messerer, Giulia Cossu

**Affiliations:** a Division of Neurosurgery, Department of Clinical Neurosciences, University Hospital of Lausanne and University of Lausanne, Lausanne, Switzerland; b Department of Laboratory Medicine and Pathology, Institute of Pathology, University of Lausanne, Lausanne, Switzerland; c Unit of Pathology, Department of Medicine and Technological Innovation, University of Insubria, Varese, Italy; d Service of Endocrinology Diabetes and Metabolism, University Hospital of Lausanne and University of Lausanne, Lausanne, Switzerland

**Keywords:** PD-L1, Pit-NETs, Pituitary neuroendocrine tumors, Pituitary adenoma, Transcription factors, 2022 WHO classification of PitNETs

## Abstract

**Introduction:**

and research question: Prognostic factors to predict the behavior of pituitary neuroendocrine tumors (PitNET) are scarce. PD-L1 expression was associated with prognosis in other neuroendocrine neoplasms and we analyzed PD-L1 expression in PitNET, according to the 2022 WHO classification.

**Material and methods:**

A retrospective analysis was performed. Immunohistochemistry was used to define PD-L1 expression, which was quantified as TPS (tumor proportion score). The primary outcome was to assess the correlation between PD-L1 expression and transcription factors (TF), namely T-pit, Pit-1, SF-1 and GATA-3. As secondary outcomes, we evaluated the association between PD-L1 expression and proliferation indexes.

**Results:**

Eighty-eight patients were included. The largest group belonged to the SF-1-lineage (48%), followed by tumors of the Pit-1 lineage (32%) and T-pit lineage (17%). PD-L1 expression was associated with Pit-1 expression (p < 0.001) and with the somatotroph, lactotroph and mammosomatotroph subgroups. A TPS ⩾35% showed a 100% sensitivity for the mammosomatotroph subtype, while the optimal cut-off point was 20% for somatotroph and 15% for lactotroph tumors. PD-L1 expression was negatively associated with SF-1 and GATA3 expression(p < 0.001), with an optimal cut-point ≤5%. No association was found between PD-L1 expression and immunohistochemical proliferative factors but PD-L1 expression was associated with female sex and a younger age at diagnosis.

**Conclusion:**

PD-L1 expression was associated with PIT-1 lineage, while it was downregulated in SF-1-lineage tumors. No correlation was found with proliferative factors. The role of PD-L1 expression in determining the biological behavior of PitNET remains debated and larger studies are necessary to further confirm these findings.

## Highlights

• Prognostic factors to predict PitNET's behaviors are scarce.
• PD-L1 was highly expressed in Pit-1 PitNET and in somatotroph and lactotroph types.
• PD-L1 was negatively associated with SF-1 and GATA3 expression.
• PD-L1 expression was associated with female sex and a younger age at diagnosis.
• No association was found with immunohistochemical proliferative factors.

## 1 Introduction

Pituitary neuroendocrine tumors (PitNETs) are one of the most frequent intracranial tumors (Molitch, 2017). The most recent World Health Organization (WHO) classification of pituitary tumors was published in 2022 and is based on a detailed histological subtyping according to the tumor cell lineage, cell type, and related characteristics (Asa et al., 2022).

In the past years, several works investigated potential prognostic factors to predict the clinical behavior of these tumors, as it is difficult to anticipate their evolution at diagnosis (Raverot et al., 2017). Patients with aggressive tumors may be refractory to standard surgical and oncological treatments and the prediction of patient's response to the different therapies is not yet possible, even after careful histopathological analysis (Ng et al., 2021).

Programmed death-ligand 1 (PD-L1) expression was related to aggressive behavior in lung tumors (Ghosh et al., 2021; Luo et al., 2021; Zhang et al., 2019) and melanoma (Yu et al., 2016; Kaunitz et al., 2017) and in other systemic neuroendocrine tumors (Rosner et al., 2021; Sampedro-Nunez et al., 2018; Multone et al., 2024). Nevertheless, its significance in determining the biological behavior of PitNETs remains debated (Cossu et al., 2023; Wang et al., 2018; Mei et al., 2016). We recently showed that a higher PD-L1 expression was associated with proliferative grades at Trouillas’ classification and with an increased p53 expression (Cossu et al., 2023). PD-L1 expression was also significantly higher in somatotroph tumors, and lower in gonadotroph tumors according to the 2017 WHO classification (Cossu et al., 2023; Turchini et al., 2021). The aim of our study is to analyze the correlation between PD-L1 expression and the 2022 WHO classification of PitNETs (Asa et al., 2022), to evaluate if new associations can be found.

## 2 Material and methods

We analyzed a consecutive series of adult patients with PitNET, operated at the Neurosurgical Department of the University Hospital of Lausanne, in Switzerland, between May 2020 and December 2023. An ethical approval was obtained before starting the study (CER-VD, 2020-01338) and the research was conducted in accordance with the declaration of Helsinki.

The primary outcome was to assess the correlation between PD-L1 expression and transcription factors (TFs), namely T-pit, Pit-1, SF-1 and GATA-3. As secondary outcomes, we evaluated the association between PD-L1 expression and the proliferation indexes such as Ki-67, number of mitoses and with p53. Only patients with a pathological analysis according to the 2022 WHO classification and with sufficient tissue for reliable PD-L1 expression analysis were included.

Two pathologists (EH & JPB) independently analyzed the samples and examined immunostaining and expression of PD-L1. All samples were fixed in buffered formalin and processed to paraffin. Staining with hematoxylin–eosin was performed for morphologic evaluation and to evaluate the presence of mitoses and necrosis. Immunohistochemical (IHC) analysis was performed using the Ventana Benchmark XT autostainer (Ventana Medical System, Tucson, AZ, USA) for hormonal expression, PD-L1 expression (clone SP263), Ki-67/MIB1 and p53. The specific presence of TF such as Pit-1, T-pit, SF-1 and GATA-3 was analyzed to classify the tumor according to the 2022 WHO classification.

The rate of PD-L1 expression was evaluated as tumor proportion score (TPS) by estimating the percentage of neoplastic cells showing membranous staining. Staining was graded linearly from 0% to 100% of cells marking. Less than 1% of cells staining was considered negative, while staining value in between 1% and 100% were scored as positive.

### 2.1 Statistical analysis

Continuous variables are presented as mean ± standard deviation (SD), and categorical variables as number and percentage.

We assessed the association between the predictor TPS and the outcome variables using logistic regression models for binary outcomes and linear regression models for continuous or ordered categorical outcomes. Monotonicity was checked using Lowess smoother for binary outcomes and scatter plots for continuous or ordered categorical outcomes. For those outcomes showing evidence of an association with TPS, we calculated the optimal cut-off point for their prediction by maximizing the product of the sensitivity and the specificity according to Liu method (Liu, 2012) in Stata v18 software (StataCorp, College Station, TX, USA).

## 3 Results

We gathered data from 100 patients: 88 patients were included while 12 patients were excluded as PD-L1 analysis was not performed. Mean age at time of surgery was 52.1 years old (SD±18 years) and 45 patients were women (51%). Seventy-eight patients underwent a first surgery (88.6%), while 10 had recurrent tumors (11.4%).

Clinical presentation is summarized in Table 1. Thirty-one patients (35%) presented a symptom or clinical sign of hormonal hypersecretion, as detailed in Table 1.

**Table 1:** The epidemiological and clinical data of the population included are resumed.

Data	Total patients N = 88 (%)
Average age in years (s.d.)	52.1 (18.0)
Gender	
- Female	− 45/88 (51)
- Male	− 43/88 (49)
Clinical presentation	
- Visually impaired	− 39/88 (44)
- Hormonal hypersecretion symptom	− 31/88 (35)
- Headache	− 24/88 (27)
- Apoplexy	− 7/88 (8)
Biological lab results	
- Stalk effect	− 17/88 (19)
- Partial hypopituitarism	− 27/88 (31)
- Total panhypopituitarism	− 2/88 (2)
Surgery	
- First surgical procedure	− 78/88 (89)
- Recurrence	− 10/88 (11)

PD-L1 was expressed in 40 tumor specimens (45%). Its expression could vary from 2% to 100% with only one patient expressing a TPS score of 100%.

The distribution of PitNET according to the 2022 WHO classification is detailed in Table 2.

**Table 2:** Our surgical cohort was stratified according to the 2022 WHO classification and to PD-L1 expression.

	PitNET type	TF	Hormones found	Results (%)	PD-L1 (%)		P value
				N = 88	Negative: TPS 0% N = 48	Positive: TPS>1% N = 40	
*PIT1-lineage PitNETs*				*28/88 (32)*	*5/48 (10)*	*23/40 (57,5)*	*<0.001*
1	Somatotroph tumors	PIT1	GH	13/88 (15)	3/48 (6)	10/40 (25)	0,005
2	Lactotroph tumors	PIT1, ER α	PRL	6/88 (7)	1/48 (2)	5/40 (12)	0,065
3	Mammosomatotroph tumors	PIT1, ER α	GH (predominant),	6/88 (7)	0 (0)	6/40 (15)	0,012
			PRL, α-subunit				
4	Thyrotroph tumors	PIT1, GATA3	α-subunit, β TSH	0 (0)	0 (0)	0 (0)	
5	Mature plurihormonal PIT1-lineage tumor	PIT1, ER α, GATA3	Predominant GH expression	1/88 (1)	0 (0)	1/40 (2)	
			Variable PRL, β TSH, α-subunit				
6	Immature PIT1-lineage tumor	PIT1, (ER α, GATA3)	Variable and focal staining of no or several hormone(s)	1/88 (1)	1/48 (2)	0 (0)	
7	Acidophil stem cell tumor	PIT1, ER α	PRL predominant	1/88 (1)	0 (0)	1/40 (2)	
			GH focal/variable				
8	Mixed somatotroph and lactotroph tumor	PIT1, ER α	2 components:	0 (0)	0 (0)	0 (0)	
			1) GH, α-subunit				
			2) PRL				
*TPIT-lineage PitNETs*				*15/88 (17)*	*7/48 (15)*	*8/40 (20)*	*0,89*
9	Corticotroph tumors	TPIT	ACTH	15/88 (17)	7/48 (15)	8/40 (20)	0,89
			Other POMC derivates				
*SF1-lineage PitNETs*				*42/88 (48)*	*34/48 (71)*	8/40 (20)	<0.001
10	Gonadotroph tumors	SF1, ER α, GATA3	α-subunit, βFSH, βLH or none	42/88 (48)	34/48 (71)	8/40 (20)	<0,001
PitNETs with no distinct cell lineage				*3/88 (3)*	*2/48 (4)*	*1/40 (2)*	*0,089*
11	Plurihormonal tumor	Multiple combination	Multiple combinations	0 (0)	0 (0)	0 (0)	
12	Null cell tumor	None	None	3/88 (3)	2/48 (4)	1/40 (2)	0,89

Legend.

PitNET(s): Pituitary neuroendocrine tumor(s).

TF: transcription factor(s).

PD-L1: Programmed death-ligand 1.

TPS: Tumor Proportion Score; PIT-1: pituitary-specific positive transcription factor 1.

TPIT: T-box transcription factor.

SF-1: The steroidogenic factor 1 transcription factor.

GATA-3: GATA binding protein 3.

ER α: Estrogen receptor alpha; GH: growth hormone; PRL: prolactine hormone; TSH: Thyroid Stimulating Hormone; LH: Luteinizing hormone.

FSH: follicle stimulating hormone.

ACTH: Adrenocorticotropic hormone.

POMC: Proopiomelanocortin.

The largest group of our cohort belonged to the SF-1-lineage (42/88, 48%), followed by tumors of the Pit-1 lineage, with 28 cases (32%). T-pit lineage was found in 15 cases (17%).

When crossmatching results of PD-L1 expression with the expression of TF and PitNETs hormonal subtypes (Table 2).

**Fig. 1:**
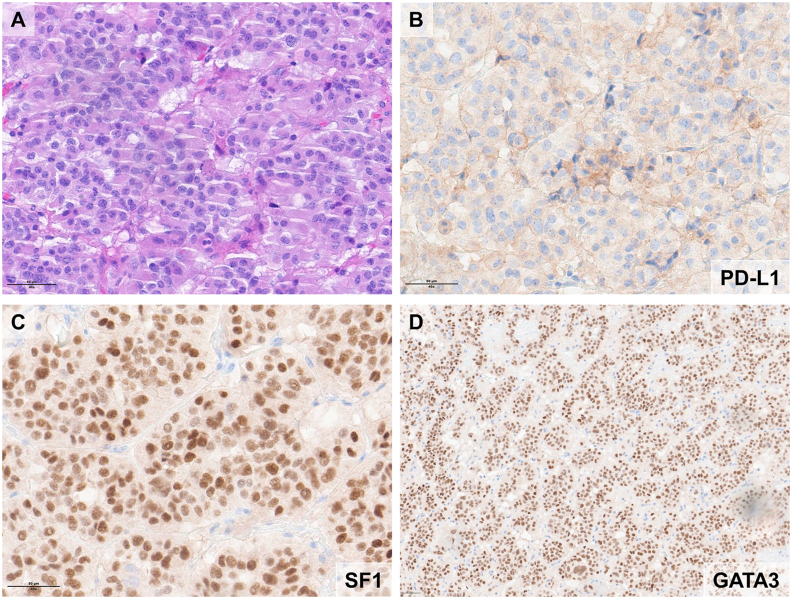
A hematoxylin and eosin staining (panel A) and immunohistochemical staining of a PitNET of SF-1 and GATA-3 lineage is illustrated (panels C and D respectively). PD-L1 staining is very low (panel B).
- Inversely, 23 out of the 28 cases expressing Pit-1, were PD-L1 positive (82%, p < 0.001). Somatotroph tumors were the most frequent subgroup in our cohort (13/88, 15%) and we found a positive association with PD-L1 expression (10/13 cases, 77%, p = 0.005). A similar association was found with the lactotroph and mammosomatotroph tumors group: 5 out of 6 lactotroph PitNET were PD-L1 positive (83%, p = 0.065) and all the 6 in the mammosomatotroph group (100%, p = 0.012) (Fig. 2).

- Seventy-one percent (34/48) of samples not expressing PD-L1 belonged to the SF-1-lineage (gonadotroph tumors). Considering only the SF-1-lineage, 81% of patients (34/42) did not express PD-L1, with a negative statistical association between SF-1 and PD-L1 expression (p < 0.001)*.* Similarly, when GATA-3 was expressed, PD-L1 was negative in 34 out of 43 samples (79%) (p < 0.001, Fig. 1).

**Fig. 2:**
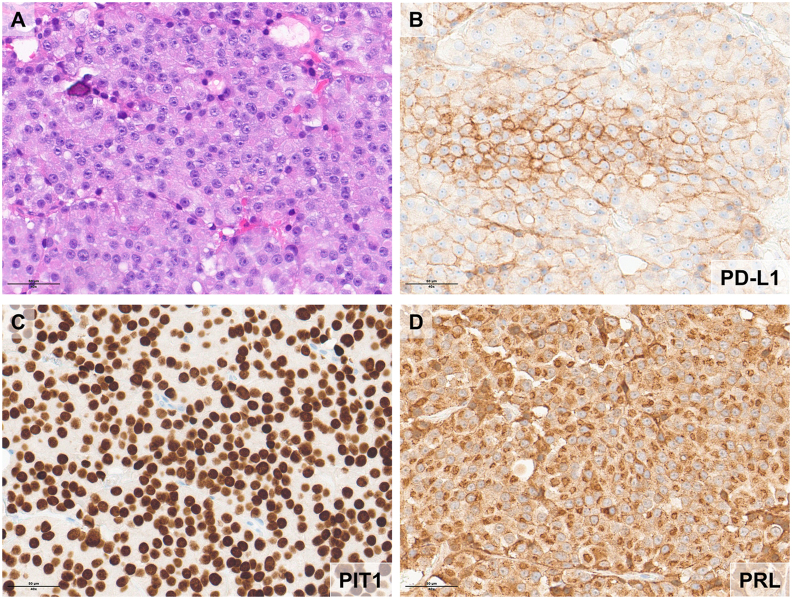
A hematoxylin and eosin staining (panel A) and immunohistochemical staining of a PitNET of PIT1 lineage is illustrated (panels C), expressing prolactin (panel D). PD-L1 expression was >30% (panel B).
- In the group expressing T-pit, there was an unspecific distribution of PD-L1 expression, and no significant association was found (p = 0,89)

**Fig. 3:**
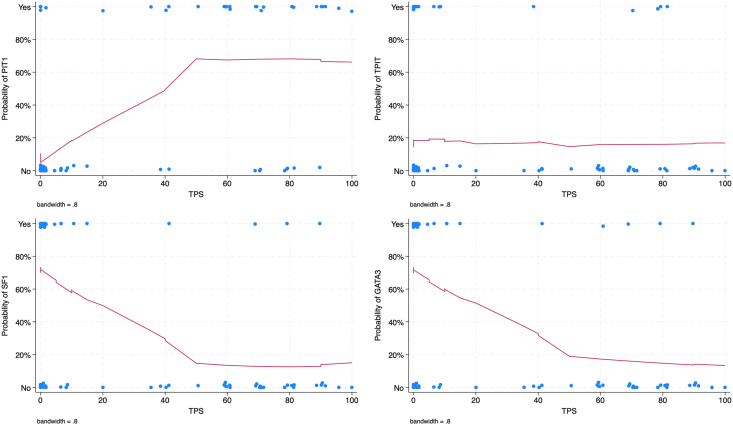
Graphical representation of PD-L1 expression, expressed as a continuous parameter (tumor proportion score or TPS), according to the presence of transcription factors, namely PIT-1, T-PIT, SF-1, GATA-3. If PD-L1 expression was elevated, the probability of PIT-1 expression was also high, while the relationship was inverse for SF-1 or GATA-3 expression.

We used a logistic regression model to evaluate the association between TPS as a continuous parameter and TF expression (Fig. 3). TPS was positively correlated with Pit-1 and with the PitNET subtypes somatotroph, lactotroph and mammosomatotroph tumors. Inversely, TPS was contrarywise correlated with SF-1, GATA3, and with gonadotroph tumors.

We established the best cut-off point of TPS to maximize sensitivity and specificity values for the different TF and PitNET subtypes (Table 3).

- The optimal cut-off point of TPS was 5% or lower for tumors expressing SF-1 and GATA3 or for gonadotroph tumors.
- It was 15% for both Pit-1 lineage and lactotroph tumors,
- For somatotroph tumors, the cut-point was at 20%, while for the mammosomatotroph PitNET it was 35%, as TPS ≥ of 35% showed a 100% sensitivity for the mammosomatotroph subtype.

**Table 3:** Optimal cut points of PD-L1 expression according to the expression of specific transcription factor and subtypes of PitNETs, while maximizing the product of the sensitivity and the specificity.

Outcome	Optimal TPS cut-point	Sensitivity	Specificity	AUC
**Transcriptions factors**				
PIT1	≥15%	0,82	0,83	0,83

SF1	≤5%	0,86	0,67	0,77
GATA3	≤5%	0,84	0,67	0,75

**Tumors**				
Mammosomatotroph	≥35%	1,00	0,70	0,85
Somatotroph	≥20%	0,77	0,71	0,74
Lactotroph	≥15%	0,83	0,66	0,75

Gonadotroph	≤5%	0,86	0,67	0,77

When crossmatching PD-L1 expression to demographic, clinical and proliferative factors (Table 4), PD-L1 expression was associated with female sex (29/40, 72%, p < 0.001) and younger age (mean age = 45.1, p < 0.001). Recurrent tumors seem to express less PD-L1 (10 cases; not statistically significant) but we should consider that 6 of them belonged to SF-1 lineage. Radiological features were not included in our analysis as they were largely described in a previous cohort (Cossu et al., 2023).

**Table 4:** Analysis of the association between PD-L1 expression and demographic and proliferative factors.

	Patients	PD-L1 TPS 0%	PD-L1 TPS >1%	P value^a^
	N = 88	N = 48	N = 40	
**Average age in years (s.d.)**	52.1 (18.0)	58.0 (15.0)	45.1 (19.0)	<0.001
**Male sex**	49% (43/88)	67% (32/48)	28% (11/40)	<0.001
**Female sex**	51% (45/88)	33% (16/48)	72% (29/40)	<0.001
**Surgery**				0.086
First procedure	89% (78/88)	83% (40/48)	95% (38/40)	
Recurrence	11% (10/88)	17% (8/48)	5% (2/40)	
**Apoplexy at presentation**	8% (7/88)	12% (6/48)	2% (1/40)	0.084
**IP MIB1/Ki-67**				0.16
0	44% (39/88)	52% (25/48)	35% (14/40)	
1	30% (26/88)	29% (14/48)	30% (12/40)	
1.5–10	26% (23/88)	19% (9/48)	35% (14/40)	
**Ki-67** ≥ **3%**	5% (4/88)	4% (2/48)	5% (2/40)	0.85
**Mitoses** ≥ **2/10**	3% (3/88)	2% (1/48)	5% (2/40)	0.45
**Necrosis**	8% (7/88)	10% (5/48)	5% (2/40)	0.35

a P values are from the Independent T test for the variable Age, and from the Chi-squared test for the remaining variables.

Concerning the analysis of proliferative factors at immunohistochemistry, Ki67 was ≥ of 3% in only 4 patients. Mitoses (more than 2 per 10 HPF) were found in 3 cases while necrosis were found in 7 cases. p53 was negative in 100% of patients. None of these results showed a significant correlation to PD-L1 expression.

## 4 Discussion

PD-L1 is a membrane protein expressed on the surface of macrophages under normal conditions. It binds to the PD-1 receptor on lymphocytes T cells membranes and modulates their activity (Parvez et al., 2023). Several cancer cells may harbor PD-L1, thus inactivating T lymphocytes, and consequently down-regulating one of the immune system defense mechanisms against tumor cells. PD-1/PD-L1 binding therefore plays a central role in the efficiency of the immune system in recognizing tumor cells and blocking tumor development (Parvez et al., 2023; Chen et al., 2020).

The pathogenesis of PitNETs is a complex process that involves abnormal transcriptomic changes, among other intrinsic and extrinsic drivers, that result in cell cycle dysregulation, loss of tumor suppressor factors, and signaling defects (Jiang and Zhang, 2013). Over the last two decades, classification frameworks of pituitary tumors have changed dramatically, reflecting improvements in understanding of tumor biology (Mete and Lopes, 2017). The 5th and last edition of the WHO classification published in 2022 (Asa et al., 2022), is based on TF and the nomenclature “PitNET” was preferred to the term “pituitary adenoma”. Several works on PitNETs and specific TF have been published recently and we further investigated the specific association between PD-L1 expression and TF expression in these tumors. We found that PitNET of SF-1 lineage generally do not express PD-L1, while tumors of PIT-1 lineage are mainly positive. These results corroborate the precedent evaluation of our team based on hormonal expression (Cossu et al., 2023) and the findings from Turchini et al. (2021). Elevated PD-L1 expression in Pit-1-lineage tumors, particularly in somatotroph PitNET, could indicate a greater propensity to evade the immune response. Indeed, an elevated PD-L1 expression could explain why some Pit-1 lineage tumors present more aggressive clinical characteristics (Luo et al., 2023; Uraki et al., 2020). Luo et al. (2023) demonstrated how PD-L1 expression in Pit-1 lineage PitNET can be correlated with tumor volume and cavernous sinus invasion. However, these results remain controversial: in the present series we did not consider radiological features as we focused only on histopathological analysis, but a previous publication from our group failed to show a correlation between PD-L1 expression and cavernous sinus invasion, tumor size, bone erosion or dura mater invasion (Cossu et al., 2023). Blockade of the PD-1/PD-L1 immune checkpoint with immunotherapies could potentially represent a promising alternative treatment for patients presenting aggressive Pit-1-lineage PitNETs with high PD-L1 expression and not responding to conventional treatments (Feola et al., 2022; Dai et al., 2020).

Gonadotrophs tumors, belonging to SF-1 lineage, are the most common non-functioning PitNET. In our cohort, these tumors were mainly PD-L1 negative, and we found an optimal cut-point of TPS ≤5% and this finding was consistent with previous works (Cossu et al., 2023; Wang et al., 2018; Mei et al., 2016; Turchini et al., 2021). Gonadotrophs tumors have less tumor-infiltrating lymphocytes (Wang et al., 2018; Mei et al., 2016) and fewer genomic alterations (Song et al., 2016): those findings may suggest that these tumors might not respond favorably to immunotherapies including PD-1/PD-L1 inhibitors (Ilie and Raverot, 2020). These targeted therapies, such as pembrolizumab and nivolumab, could offer new therapeutic options, specifically for PitNETs resistant to conventional therapies (Feola et al., 2022; Serioli et al., 2023). Currently, there are running trials on combined immunotherapies for aggressive pituitary tumors (and pituitary carcinomas), including PitNET of gonadotroph origin (Ilie and Raverot, 2020), but their clinical efficacy is still debated (Ilie et al., 2022; Even-Zohar and Greenman, 2022). For sure, an accurate identification of the subgroups of PitNET patients who might benefit from these target therapies is the first critical step in the risk-benefit assessment (Su et al., 2020).

Our analysis showed that patients having a positive PD-L1 expression were more frequently women and younger than those with no expression. This could suggest that PD-L1 expression may be associated with age-related factors, hormonal or genetic factors. No specific studies about sex- or age-related differences have been published in patients with PitNETs but higher PD-L1 levels were found in non-small cell lung carcinomas of patients with male sex (Rodriguez-Lara et al., 2023) and younger age (Cooper et al., 2015). However, further dedicated studies are required to confirm these findings in populations of PitNET.

PD-L1 expression in recurrent tumors is variable in literature. In our series PD-L1 had a low expression and this could be partly attributed to the predominance of tumors belonging to the SF-1 lineage. Similar results were reported by Dai et al. (2020). On the contrary, Shi et al. found high PD-L1 expression in recurrent tumors (Shi et al., 2023) and this could, at least in part, be attributed to the young age of their surgical series.

In our cohort we could not confirm the association between PD-L1 expression and the number of mitoses or Ki-67 expression. Subsequently, the role of PD-L1 in predicting tumor behavior is not yet fully understood and requires further investigation (Cossu et al., 2023; Wang et al., 2018; Mei et al., 2016; Suteau et al., 2020).

## 5 Limitations

Measurement biases due to the small sample size may have occurred and larger studies are mandatory to corroborate our results. The number of patients expressing proliferative factors was low and the power of our analysis was limited. Furthermore, the search for PD-L1 expression in PitNET has increased over the last decade but its immunohistochemistry interpretation may be challenging, as there is a lack of consensus about assessment criteria, antibodies used for immunohistochemistry measurements, along with inter-observer variability (Paver et al., 2021; Gompelmann et al., 2024; Chang et al., 2019). Furthermore, heterogeneity in evaluating the same sample and between different evaluations was described (O'Malley et al., 2019; Scognamiglio et al., 2016).

As previously mentioned, we did not include correlation analysis between PD-L1 expression and radiological features of the tumors in this paper. All these factors can lead to discrepancy in results and therefore to limitations in the interpretation of PD-L1 expression (Lopes-Pinto et al., 2024).

## 6 Conclusion

In our study, Pit-1 lineage tumors presented a positive association with PD-L1 expression, while an inverse relationship was found for SF-1 and GATA3 lineage tumors. Somatotroph tumors expressed PD-L1 with an optimal cut-point of TPS ≥20%, lactotroph tumors ≥15% and mammosomatotroph ≥35%. Inversely, gonadotroph tumors didn't express PD-L1 (optimal TPS cut-point ≤5%). No association was detected between PD-L1 expression and proliferative factors. PD-L1 expression was associated with female sex and a younger age.

The possible role of PD-L1 expression in determining the biological behavior of PitNET remains debated, as its expression seems to be mainly related to the expression of specific transcription factors.

## Author contribution

Conceptualization, E.H., M.M., and G.C.; methodology, E.H., M.M. and G.C.; validation, all authors; formal analysis, E.H.; investigation, E.H, E.H, J.P.B., M.M., and G.C.,; resources, E.H. and J.P.B.; data curation, S.L.R., E.H. and J.P.B and J.P.B.; writing—original draft preparation, E.H., and G.C.; writing—review and editing, E.H., F.S. N.P.,R.T.D., M.M. and G.C; visualization, E.H., M.M. and G.C.,; supervision, R.T.D. and M.M.; project administration, G.C. and M.M.; funding acquisition, G.C. and M.M.

All authors have read and agreed to the published version of the manuscript.

## Institutional review board statement

The study was conducted according to the guidelines of the Declaration of Helsinki and approved by the Local Ethics Committee CER-VD (Project-ID, 2020-01338, the July 27, 2020).

## Data availability statement

The data are available on simple request at the first author.

## Funding

This project was partly financed by the Brihaye EANS Research Grant.

## Declaration of competing interest

The authors declare the following financial interests/personal relationships which may be considered as potential competing interests: Giulia Cossu reports financial support was provided by The European Association of Neurosurgical Societies. If there are other authors, they declare that they have no known competing financial interests or personal relationships that could have appeared to influence the work reported in this paper.
